# *catena*-Poly[[tetra­kis­(hexa­methyl­phospho­ramide-κ*O*)bis­(nitrato-κ^2^ *O*,*O*′)samarium(III)] [silver(I)-di-μ-sulfido-tungstate(VI)-di-μ-sulfido]]

**DOI:** 10.1107/S1600536811035574

**Published:** 2011-09-14

**Authors:** Jinfang Zhang

**Affiliations:** a Molecular Materials Research Center, Scientific Research Academy, School of Chemistry and Chemical Engineering, Jiangsu University, Zhenjiang 212013, People’s Republic of China

## Abstract

The Sm atom in the cation of the title salt, {[Sm(NO_3_)_2_(C_6_H_18_N_3_OP)_4_][AgS_4_W]}_*n*_, is coordinated by eight O atoms derived from two chelating nitrate anions, and four hexamethylphos­phoramide ligand, defining a distorted square-antiprismatic geometry. The anions self-assemble into polymeric chains *via* W—S—Ag bridges having a [AgS_4_W] repeat unit; the W—Ag—W and Ag—W—Ag angles are 161.657 (17) and 153.978 (9)°, respectively. The title complex is isostructural with the Y, Yb, Eu, Nd, La and Dy isomorphs.

## Related literature

For one-dimensional Mo(W)/S/Ag anionic polymers and their properties, see: Niu *et al.* (2004 ▶); Zhang, Song & Wang (2007 ▶). For the structure of isotypic Y, Yb, Eu, Nd, La and Dy complexes, see: Zhang, Cao *et al.* (2007 ▶); Zhang (2011 ▶); Cao *et al.* (2007 ▶); Zhang, Qian *et al.* (2007 ▶); Tang, Zhang & Zhang (2008 ▶); Tang, Zhang, Zhang & Lu (2008 ▶); Zhang (2010 ▶).
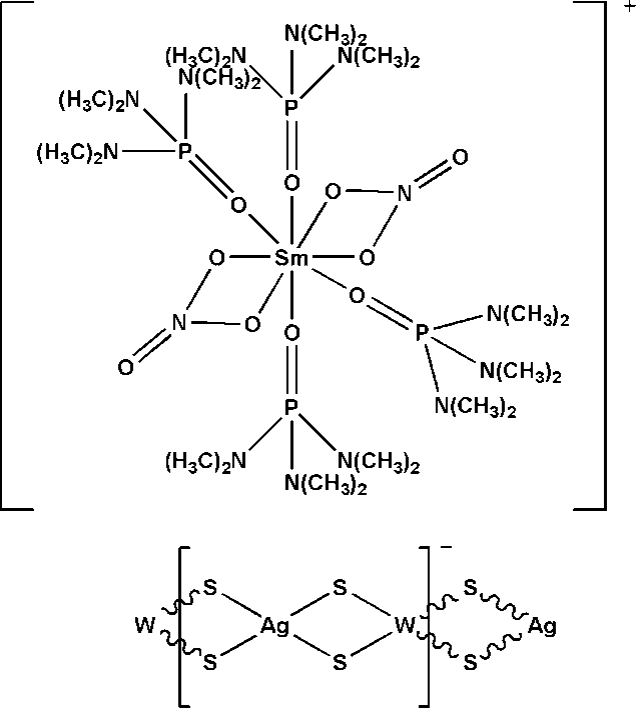


## Experimental

### 

#### Crystal data

[Sm(NO_3_)_2_(C_6_H_18_N_3_OP)_4_][AgS_4_W]
*M* *_r_* = 1411.19
Monoclinic, 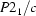

*a* = 15.817 (3) Å
*b* = 29.768 (6) Å
*c* = 11.372 (2) Å
β = 91.03 (3)°
*V* = 5353.5 (18) Å^3^
*Z* = 4
Mo *K*α radiation
μ = 3.92 mm^−1^
*T* = 293 K
0.2 × 0.16 × 0.1 mm

#### Data collection

Rigaku Saturn724+ diffractometer
Absorption correction: multi-scan (*CrystalClear*; Rigaku, 2007 ▶) *T*_min_ = 0.476, *T*_max_ = 0.676
24655 measured reflections
9738 independent reflections
9057 reflections with *I* > 2σ(*I*)
*R*_int_ = 0.022
Standard reflections: 0

#### Refinement

*R*[*F* ^2^ > 2σ(*F* ^2^)] = 0.031
*wR*(*F* ^2^) = 0.078
*S* = 1.03
9738 reflections
532 parameters
H-atom parameters constrained
Δρ_max_ = 1.16 e Å^−3^
Δρ_min_ = −1.06 e Å^−3^

### 

Data collection: *CrystalClear* (Rigaku, 2007 ▶); cell refinement: *CrystalClear*; data reduction: *CrystalClear*; program(s) used to solve structure: *SHELXTL* (Sheldrick, 2008 ▶); program(s) used to refine structure: *SHELXTL*; molecular graphics: *SHELXTL*; software used to prepare material for publication: *SHELXTL*.

## Supplementary Material

Crystal structure: contains datablock(s) I, global. DOI: 10.1107/S1600536811035574/rz2631sup1.cif

Structure factors: contains datablock(s) I. DOI: 10.1107/S1600536811035574/rz2631Isup2.hkl

Additional supplementary materials:  crystallographic information; 3D view; checkCIF report

## Figures and Tables

**Fig. 1.**
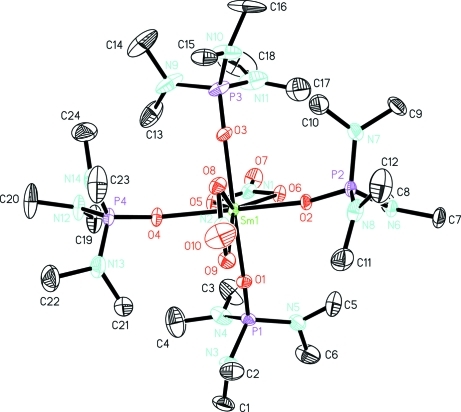
The molecular structure of the cation in the title compound, with 30% probability displacement ellipsoids. All H atoms have been omitted.

**Fig. 2.**
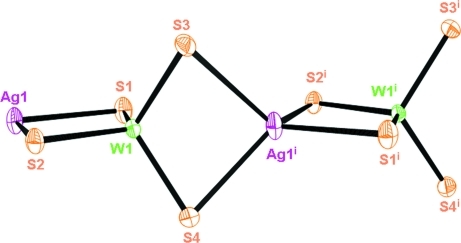
The structure of a portion of the anionic chain in the title compound, with 30% probability displacement ellipsoids. Symmetry code: (i) x, -y + 1/2, z - 1/2.

## supplementary crystallographic information

### Comment

One-dimensional Mo(W)/S/Ag anionic polymers have attracted much attention for their configurational isomerism (Niu *et al.*, 2004) and unique properties as functional materials, such as third-order nonlinear optical (NLO) materials (Zhang, Song & Wang, 2007). Different solvent-coordinated rare-earth cations proved effective to obtain various configurations of anionic chains (Niu *et al.*, 2004). The title compound {[Sm(hmp)_4_(NO_3_)_2_][WS_4_Ag]}_n_ (hmp = hexamethylphosphoramide) with a wave-like anionic chain was prepared by following such route using Sm(III)-hmp complex as counterion.

The title complex is isostructural with Y (Zhang, Cao *et al.*, 2007; Zhang, 2011), Yb (Cao *et al.*, 2007), Eu (Zhang, Qian *et al.*, 2007), Nd (Tang, Zhang & Zhang, 2008), La (Tang, Zhang, Zhang & Lu, 2008) and Dy (Zhang, 2010) isomorphs. The Sm atom in the cation is coordinated by eight O atoms from two nitrate and four hmp ligands. In possession of two nitrate ligands, the cation in the title compound is univalent (Fig. 1), which leads to an anionic chain with a univalent repeat unit. As illustrated in Fig. 2, the anionic chain in the title compound has a distorted linear configuration with W—Ag—W and Ag—W—Ag angles of 161.657 (17) and 153.978 (9) °, respectively.

### Experimental

1 mmol AgI was added to a solution of [NH_4_]_2_WS_4_ (1 mmol in 10 ml hmp) with thorough stirring for 30 minutes. The solution underwent an additional stirring for two minute, then 0.5 mmol Sm(NO_3_)_3_.6H_2_O was added. After filtration the orange filtrate was carefully laid on the surface with 12 ml *i*-PrOH. Orange block crystals were obtained after about one week.

### Refinement

H atoms were positioned geometrically and refined with riding model, with *U*_iso_ = 1.5*U*_eq_ and C—H = 0.96 Å.

### Crystal data

**Table:**

[Sm(NO_3_)_2_(C_6_H_18_N_3_OP)_4_][AgS_4_W]	*F*(000) = 2804.0
*M**_r_* = 1411.19	*D*_x_ = 1.751 Mg m^−^^3^
Monoclinic, *P*2_1_/*c*	Mo *K*α radiation, λ = 0.71073 Å
Hall symbol: -P 2ybc	Cell parameters from 22427 reflections
*a* = 15.817 (3) Å	θ = 3.0–29.1°
*b* = 29.768 (6) Å	µ = 3.92 mm^−^^1^
*c* = 11.372 (2) Å	*T* = 293 K
β = 91.03 (3)°	Block, orange
*V* = 5353.5 (18) Å^3^	0.2 × 0.16 × 0.1 mm
*Z* = 4	

### Data collection

**Table:**

Rigaku Saturn724+ diffractometer	9738 independent reflections
Radiation source: fine-focus sealed tube	9057 reflections with *I* > 2σ(*I*)
graphite	*R*_int_ = 0.022
dtprofit.ref scans	θ_max_ = 25.4°, θ_min_ = 3.0°
Absorption correction: multi-scan (*CrystalClear*; Rigaku, 2007)	*h* = −19→14
*T*_min_ = 0.476, *T*_max_ = 0.676	*k* = −35→35
24655 measured reflections	*l* = −13→12

### Refinement

**Table:**

Refinement on *F*^2^	Primary atom site location: structure-invariant direct methods
Least-squares matrix: full	Secondary atom site location: difference Fourier map
*R*[*F*^2^ > 2σ(*F*^2^)] = 0.031	Hydrogen site location: inferred from neighbouring sites
*wR*(*F*^2^) = 0.078	H-atom parameters constrained
*S* = 1.03	*w* = 1/[σ^2^(*F*_o_^2^) + (0.0345*P*)^2^ + 16.4718*P*] where *P* = (*F*_o_^2^ + 2*F*_c_^2^)/3
9738 reflections	(Δ/σ)_max_ = 0.001
532 parameters	Δρ_max_ = 1.16 e Å^−^^3^
0 restraints	Δρ_min_ = −1.06 e Å^−^^3^

### Special details

**Table:**

Geometry. All e.s.d.'s (except the e.s.d. in the dihedral angle between two l.s. planes) are estimated using the full covariance matrix. The cell e.s.d.'s are taken into account individually in the estimation of e.s.d.'s in distances, angles and torsion angles; correlations between e.s.d.'s in cell parameters are only used when they are defined by crystal symmetry. An approximate (isotropic) treatment of cell e.s.d.'s is used for estimating e.s.d.'s involving l.s. planes.
Refinement. Refinement of *F*^2^ against ALL reflections. The weighted *R*-factor *wR* and goodness of fit *S* are based on *F*^2^, conventional *R*-factors *R* are based on *F*, with *F* set to zero for negative *F*^2^. The threshold expression of *F*^2^ > σ(*F*^2^) is used only for calculating *R*-factors(gt) *etc*. and is not relevant to the choice of reflections for refinement. *R*-factors based on *F*^2^ are statistically about twice as large as those based on *F*, and *R*- factors based on ALL data will be even larger.

### Fractional atomic coordinates and isotropic or equivalent isotropic displacement parameters (Å^2^)

**Table:**

	*x*	*y*	*z*	*U*_iso_*/*U*_eq_	
Sm1	0.737078 (14)	0.082698 (7)	0.82970 (2)	0.02352 (7)	
P1	0.69501 (9)	−0.03068 (4)	0.69384 (12)	0.0347 (3)	
P2	0.51916 (8)	0.13456 (4)	0.82656 (11)	0.0288 (3)	
P3	0.79653 (11)	0.14726 (5)	1.10222 (14)	0.0467 (4)	
P4	0.96063 (8)	0.09563 (4)	0.73301 (13)	0.0335 (3)	
O1	0.7061 (2)	0.01700 (11)	0.7267 (3)	0.0348 (8)	
O2	0.59965 (19)	0.10824 (11)	0.8257 (3)	0.0311 (8)	
O3	0.7729 (2)	0.12756 (11)	0.9874 (3)	0.0366 (8)	
O4	0.8783 (2)	0.08027 (11)	0.7821 (3)	0.0364 (8)	
O5	0.7995 (2)	0.02572 (12)	0.9719 (3)	0.0395 (9)	
O6	0.6671 (2)	0.04009 (12)	0.9942 (3)	0.0368 (8)	
O7	0.7350 (3)	0.00096 (15)	1.1273 (4)	0.0596 (12)	
O8	0.7511 (2)	0.15923 (11)	0.7355 (3)	0.0396 (9)	
O9	0.7221 (2)	0.10477 (12)	0.6171 (3)	0.0396 (9)	
O10	0.7223 (4)	0.17283 (16)	0.5515 (4)	0.0772 (16)	
N1	0.7339 (3)	0.02158 (14)	1.0343 (4)	0.0376 (10)	
N2	0.7320 (3)	0.14657 (15)	0.6320 (4)	0.0423 (11)	
N3	0.7169 (3)	−0.03673 (15)	0.5547 (4)	0.0448 (12)	
N4	0.7591 (4)	−0.06646 (16)	0.7605 (5)	0.0589 (15)	
N5	0.6019 (4)	−0.0476 (2)	0.7318 (6)	0.0703 (18)	
N6	0.4419 (2)	0.09986 (15)	0.8524 (4)	0.0338 (10)	
N7	0.5222 (3)	0.17346 (16)	0.9285 (5)	0.0464 (12)	
N8	0.5024 (3)	0.15940 (18)	0.7019 (4)	0.0485 (12)	
N9	0.8986 (5)	0.1380 (2)	1.1266 (7)	0.090 (2)	
N10	0.7788 (5)	0.19980 (17)	1.1016 (5)	0.0719 (19)	
N11	0.7432 (6)	0.1236 (2)	1.2050 (5)	0.089 (2)	
N12	1.0359 (3)	0.07061 (17)	0.8063 (5)	0.0503 (13)	
N13	0.9587 (3)	0.0852 (2)	0.5901 (5)	0.0572 (14)	
N14	0.9844 (3)	0.14859 (16)	0.7372 (5)	0.0534 (14)	
C1	0.7225 (5)	−0.0823 (2)	0.5013 (6)	0.067 (2)	
H1A	0.7358	−0.0796	0.4196	0.101*	
H1B	0.6693	−0.0975	0.5087	0.101*	
H1C	0.7659	−0.0993	0.5411	0.101*	
C2	0.6989 (5)	−0.0014 (2)	0.4712 (6)	0.0655 (19)	
H2A	0.7162	−0.0107	0.3944	0.098*	
H2B	0.7293	0.0253	0.4939	0.098*	
H2C	0.6393	0.0048	0.4697	0.098*	
C3	0.7518 (7)	−0.0816 (3)	0.8757 (8)	0.101 (3)	
H3A	0.7976	−0.1017	0.8942	0.152*	
H3B	0.6990	−0.0971	0.8839	0.152*	
H3C	0.7538	−0.0564	0.9284	0.152*	
C4	0.8489 (6)	−0.0704 (3)	0.7282 (9)	0.107 (3)	
H4A	0.8761	−0.0926	0.7770	0.161*	
H4B	0.8765	−0.0419	0.7395	0.161*	
H4C	0.8525	−0.0791	0.6472	0.161*	
C5	0.5332 (4)	−0.0162 (3)	0.7495 (7)	0.070 (2)	
H5A	0.4831	−0.0325	0.7701	0.105*	
H5B	0.5227	0.0004	0.6784	0.105*	
H5C	0.5482	0.0042	0.8118	0.105*	
C6	0.5771 (6)	−0.0950 (3)	0.7156 (8)	0.107 (4)	
H6A	0.5200	−0.0991	0.7408	0.160*	
H6B	0.6142	−0.1139	0.7615	0.160*	
H6C	0.5809	−0.1029	0.6341	0.160*	
C7	0.3539 (3)	0.1094 (2)	0.8175 (5)	0.0477 (15)	
H7A	0.3184	0.0849	0.8408	0.072*	
H7B	0.3500	0.1131	0.7337	0.072*	
H7C	0.3357	0.1365	0.8551	0.072*	
C8	0.4516 (4)	0.0658 (2)	0.9449 (5)	0.0459 (14)	
H8A	0.4009	0.0483	0.9490	0.069*	
H8B	0.4620	0.0804	1.0191	0.069*	
H8C	0.4984	0.0466	0.9273	0.069*	
C9	0.4536 (4)	0.1839 (2)	1.0070 (6)	0.0572 (17)	
H9A	0.4703	0.2080	1.0585	0.086*	
H9B	0.4404	0.1578	1.0529	0.086*	
H9C	0.4046	0.1927	0.9617	0.086*	
C10	0.5905 (4)	0.2073 (2)	0.9267 (7)	0.0650 (19)	
H10A	0.5838	0.2278	0.9910	0.098*	
H10B	0.5876	0.2236	0.8538	0.098*	
H10C	0.6443	0.1926	0.9341	0.098*	
C11	0.5143 (5)	0.1364 (3)	0.5944 (6)	0.079 (2)	
H11A	0.5015	0.1562	0.5299	0.118*	
H11B	0.4774	0.1108	0.5906	0.118*	
H11C	0.5719	0.1266	0.5899	0.118*	
C12	0.4619 (5)	0.2038 (3)	0.6899 (8)	0.084 (3)	
H12A	0.4582	0.2119	0.6082	0.125*	
H12B	0.4949	0.2258	0.7319	0.125*	
H12C	0.4061	0.2026	0.7217	0.125*	
C13	0.9369 (5)	0.0953 (3)	1.1123 (9)	0.096 (3)	
H13A	0.9958	0.0972	1.1340	0.144*	
H13B	0.9314	0.0861	1.0316	0.144*	
H13C	0.9096	0.0737	1.1616	0.144*	
C14	0.9560 (7)	0.1710 (4)	1.1831 (10)	0.135 (5)	
H14A	1.0115	0.1583	1.1913	0.202*	
H14B	0.9352	0.1787	1.2594	0.202*	
H14C	0.9584	0.1976	1.1355	0.202*	
C15	0.8064 (5)	0.22735 (19)	1.0012 (6)	0.0614 (19)	
H15A	0.7914	0.2582	1.0144	0.092*	
H15B	0.7790	0.2169	0.9304	0.092*	
H15C	0.8666	0.2249	0.9938	0.092*	
C16	0.7532 (8)	0.2255 (3)	1.2080 (7)	0.122 (5)	
H16A	0.7462	0.2566	1.1880	0.183*	
H16B	0.7961	0.2226	1.2683	0.183*	
H16C	0.7008	0.2138	1.2362	0.183*	
C17	0.6521 (6)	0.1215 (3)	1.1937 (8)	0.099 (3)	
H17A	0.6295	0.1070	1.2617	0.149*	
H17B	0.6367	0.1047	1.1245	0.149*	
H17C	0.6295	0.1513	1.1874	0.149*	
C18	0.7897 (10)	0.1068 (4)	1.3136 (8)	0.168 (7)	
H18A	0.7500	0.0942	1.3673	0.252*	
H18B	0.8191	0.1313	1.3509	0.252*	
H18C	0.8297	0.0841	1.2916	0.252*	
C19	1.0227 (4)	0.0278 (2)	0.8622 (7)	0.072 (2)	
H19A	1.0740	0.0184	0.9012	0.108*	
H19B	1.0067	0.0059	0.8038	0.108*	
H19C	0.9786	0.0305	0.9187	0.108*	
C20	1.1249 (4)	0.0812 (3)	0.7889 (8)	0.092 (3)	
H20A	1.1594	0.0631	0.8405	0.139*	
H20B	1.1346	0.1124	0.8059	0.139*	
H20C	1.1393	0.0752	0.7088	0.139*	
C21	0.9098 (4)	0.0469 (3)	0.5462 (7)	0.072 (2)	
H21A	0.9144	0.0451	0.4623	0.108*	
H21B	0.8515	0.0507	0.5662	0.108*	
H21C	0.9312	0.0198	0.5813	0.108*	
C22	1.0333 (5)	0.0942 (4)	0.5197 (7)	0.093 (3)	
H22A	1.0216	0.0862	0.4392	0.140*	
H22B	1.0801	0.0767	0.5492	0.140*	
H22C	1.0472	0.1255	0.5244	0.140*	
C23	0.9529 (5)	0.1811 (3)	0.6518 (9)	0.094 (3)	
H23A	0.9749	0.2103	0.6708	0.141*	
H23B	0.8922	0.1818	0.6535	0.141*	
H23C	0.9706	0.1726	0.5746	0.141*	
C24	1.0127 (6)	0.1689 (3)	0.8474 (8)	0.099 (3)	
H24A	1.0242	0.2002	0.8351	0.149*	
H24B	1.0633	0.1541	0.8750	0.149*	
H24C	0.9694	0.1657	0.9049	0.149*	
W1	0.214355 (12)	0.272009 (6)	−0.022422 (15)	0.02289 (6)	
Ag1	0.21554 (3)	0.234663 (14)	0.21751 (3)	0.04033 (11)	
S1	0.21500 (10)	0.19943 (4)	0.01873 (11)	0.0386 (3)	
S2	0.21225 (9)	0.31505 (4)	0.13519 (11)	0.0332 (3)	
S3	0.10070 (9)	0.28668 (5)	−0.12896 (12)	0.0397 (3)	
S4	0.32849 (8)	0.28805 (5)	−0.12238 (11)	0.0397 (3)	

### Atomic displacement parameters (Å^2^)

**Table:**

	*U*^11^	*U*^22^	*U*^33^	*U*^12^	*U*^13^	*U*^23^
Sm1	0.01783 (12)	0.01705 (12)	0.03568 (14)	0.00005 (9)	0.00088 (10)	0.00038 (10)
P1	0.0428 (8)	0.0251 (6)	0.0365 (7)	−0.0083 (6)	0.0102 (6)	−0.0077 (6)
P2	0.0202 (6)	0.0330 (7)	0.0333 (7)	0.0051 (5)	−0.0012 (5)	0.0043 (6)
P3	0.0650 (11)	0.0349 (8)	0.0395 (8)	−0.0128 (7)	−0.0210 (7)	0.0034 (7)
P4	0.0202 (6)	0.0308 (7)	0.0497 (8)	−0.0007 (5)	0.0042 (6)	0.0067 (6)
O1	0.038 (2)	0.0229 (17)	0.044 (2)	−0.0018 (15)	−0.0006 (16)	−0.0052 (16)
O2	0.0195 (17)	0.0330 (18)	0.0407 (19)	0.0057 (14)	−0.0002 (14)	−0.0011 (16)
O3	0.034 (2)	0.0316 (19)	0.044 (2)	−0.0028 (15)	−0.0102 (16)	−0.0045 (16)
O4	0.0193 (17)	0.0331 (19)	0.057 (2)	−0.0005 (14)	0.0044 (16)	0.0074 (17)
O5	0.0250 (19)	0.0344 (19)	0.059 (2)	−0.0006 (15)	−0.0022 (17)	0.0104 (18)
O6	0.0261 (19)	0.0362 (19)	0.048 (2)	−0.0016 (15)	−0.0019 (16)	0.0101 (17)
O7	0.058 (3)	0.064 (3)	0.057 (3)	−0.004 (2)	−0.008 (2)	0.031 (2)
O8	0.041 (2)	0.0272 (18)	0.051 (2)	−0.0057 (16)	−0.0033 (18)	0.0028 (17)
O9	0.046 (2)	0.0301 (19)	0.042 (2)	−0.0039 (16)	0.0071 (17)	0.0024 (17)
O10	0.120 (5)	0.050 (3)	0.060 (3)	−0.025 (3)	−0.013 (3)	0.034 (2)
N1	0.039 (3)	0.027 (2)	0.046 (3)	−0.0047 (19)	−0.007 (2)	0.007 (2)
N2	0.039 (3)	0.038 (3)	0.051 (3)	−0.007 (2)	0.002 (2)	0.015 (2)
N3	0.061 (3)	0.038 (3)	0.036 (2)	−0.011 (2)	0.009 (2)	−0.010 (2)
N4	0.092 (4)	0.027 (2)	0.058 (3)	0.009 (3)	0.021 (3)	0.007 (2)
N5	0.058 (4)	0.066 (4)	0.088 (4)	−0.031 (3)	0.034 (3)	−0.040 (3)
N6	0.020 (2)	0.043 (2)	0.039 (2)	0.0006 (18)	−0.0016 (18)	0.002 (2)
N7	0.037 (3)	0.042 (3)	0.061 (3)	0.006 (2)	0.006 (2)	−0.009 (2)
N8	0.034 (3)	0.070 (3)	0.041 (3)	−0.002 (2)	−0.007 (2)	0.023 (3)
N9	0.097 (5)	0.057 (4)	0.113 (6)	−0.015 (4)	−0.066 (4)	−0.008 (4)
N10	0.136 (6)	0.035 (3)	0.044 (3)	−0.009 (3)	0.006 (3)	−0.009 (3)
N11	0.149 (7)	0.069 (4)	0.051 (4)	−0.040 (5)	−0.003 (4)	0.004 (3)
N12	0.022 (2)	0.062 (3)	0.067 (3)	0.003 (2)	0.004 (2)	0.040 (3)
N13	0.037 (3)	0.083 (4)	0.052 (3)	−0.012 (3)	0.004 (2)	0.013 (3)
N14	0.035 (3)	0.040 (3)	0.085 (4)	−0.002 (2)	0.014 (3)	0.011 (3)
C1	0.090 (6)	0.053 (4)	0.059 (4)	−0.013 (4)	0.025 (4)	−0.026 (3)
C2	0.087 (5)	0.067 (5)	0.042 (4)	0.001 (4)	−0.007 (3)	−0.001 (3)
C3	0.128 (8)	0.080 (6)	0.097 (7)	0.047 (6)	0.018 (6)	0.027 (5)
C4	0.074 (6)	0.118 (8)	0.131 (9)	0.032 (6)	0.016 (6)	0.037 (7)
C5	0.039 (4)	0.074 (5)	0.097 (6)	−0.001 (3)	−0.005 (4)	0.017 (4)
C6	0.124 (8)	0.084 (6)	0.114 (7)	−0.069 (6)	0.062 (6)	−0.044 (6)
C7	0.023 (3)	0.071 (4)	0.049 (3)	0.000 (3)	−0.004 (2)	0.004 (3)
C8	0.036 (3)	0.053 (4)	0.049 (3)	0.004 (3)	0.008 (3)	0.012 (3)
C9	0.055 (4)	0.058 (4)	0.059 (4)	0.020 (3)	0.014 (3)	−0.014 (3)
C10	0.055 (4)	0.045 (4)	0.096 (6)	−0.006 (3)	0.013 (4)	−0.017 (4)
C11	0.064 (5)	0.132 (8)	0.040 (4)	−0.007 (5)	−0.010 (3)	−0.005 (4)
C12	0.064 (5)	0.078 (5)	0.109 (7)	0.017 (4)	−0.014 (5)	0.052 (5)
C13	0.066 (5)	0.075 (5)	0.144 (9)	−0.005 (4)	−0.054 (6)	0.022 (6)
C14	0.132 (10)	0.147 (10)	0.123 (9)	−0.068 (8)	−0.062 (7)	0.003 (8)
C15	0.102 (6)	0.030 (3)	0.052 (4)	−0.002 (3)	−0.001 (4)	−0.001 (3)
C16	0.246 (14)	0.060 (5)	0.061 (5)	−0.035 (7)	0.036 (7)	−0.039 (4)
C17	0.116 (8)	0.109 (7)	0.075 (6)	−0.042 (6)	0.045 (6)	−0.003 (5)
C18	0.303 (19)	0.158 (11)	0.043 (5)	0.075 (12)	0.014 (8)	0.035 (6)
C19	0.041 (4)	0.067 (5)	0.107 (6)	0.008 (3)	0.008 (4)	0.046 (4)
C20	0.029 (4)	0.124 (7)	0.124 (7)	0.003 (4)	0.005 (4)	0.075 (6)
C21	0.052 (4)	0.098 (6)	0.065 (5)	−0.004 (4)	0.004 (3)	−0.023 (4)
C22	0.061 (5)	0.160 (9)	0.060 (5)	−0.027 (5)	0.015 (4)	0.008 (5)
C23	0.053 (5)	0.057 (4)	0.174 (9)	0.010 (4)	0.020 (5)	0.060 (6)
C24	0.123 (8)	0.076 (6)	0.101 (7)	−0.049 (5)	0.037 (6)	−0.036 (5)
W1	0.02651 (11)	0.02400 (10)	0.01807 (10)	0.00269 (7)	−0.00193 (7)	−0.00178 (7)
Ag1	0.0602 (3)	0.0406 (2)	0.02010 (19)	0.00097 (19)	−0.00104 (18)	0.00200 (16)
S1	0.0613 (9)	0.0251 (6)	0.0294 (6)	0.0055 (6)	−0.0003 (6)	−0.0031 (5)
S2	0.0463 (8)	0.0273 (6)	0.0261 (6)	−0.0009 (5)	0.0009 (5)	−0.0064 (5)
S3	0.0315 (7)	0.0569 (9)	0.0306 (7)	0.0133 (6)	−0.0067 (5)	−0.0023 (6)
S4	0.0310 (7)	0.0575 (9)	0.0307 (7)	−0.0069 (6)	0.0018 (5)	−0.0040 (6)

### Geometric parameters (Å, °)

**Table:**

Sm1—O3	2.298 (3)	C6—H6A	0.9600
Sm1—O2	2.303 (3)	C6—H6B	0.9600
Sm1—O4	2.309 (3)	C6—H6C	0.9600
Sm1—O1	2.328 (3)	C7—H7A	0.9600
Sm1—O9	2.512 (4)	C7—H7B	0.9600
Sm1—O8	2.529 (3)	C7—H7C	0.9600
Sm1—O6	2.531 (3)	C8—H8A	0.9600
Sm1—O5	2.532 (4)	C8—H8B	0.9600
Sm1—N2	2.944 (5)	C8—H8C	0.9600
Sm1—N1	2.954 (4)	C9—H9A	0.9600
P1—O1	1.477 (3)	C9—H9B	0.9600
P1—N5	1.622 (5)	C9—H9C	0.9600
P1—N3	1.635 (5)	C10—H10A	0.9600
P1—N4	1.646 (6)	C10—H10B	0.9600
P2—O2	1.495 (3)	C10—H10C	0.9600
P2—N8	1.616 (5)	C11—H11A	0.9600
P2—N6	1.631 (4)	C11—H11B	0.9600
P2—N7	1.639 (5)	C11—H11C	0.9600
P3—O3	1.474 (4)	C12—H12A	0.9600
P3—N10	1.589 (6)	C12—H12B	0.9600
P3—N11	1.615 (7)	C12—H12C	0.9600
P3—N9	1.656 (7)	C13—H13A	0.9600
P4—O4	1.498 (3)	C13—H13B	0.9600
P4—N14	1.621 (5)	C13—H13C	0.9600
P4—N12	1.622 (5)	C14—H14A	0.9600
P4—N13	1.654 (6)	C14—H14B	0.9600
O5—N1	1.274 (6)	C14—H14C	0.9600
O6—N1	1.269 (5)	C15—H15A	0.9600
O7—N1	1.223 (6)	C15—H15B	0.9600
O8—N2	1.268 (6)	C15—H15C	0.9600
O9—N2	1.265 (6)	C16—H16A	0.9600
O10—N2	1.211 (6)	C16—H16B	0.9600
N3—C2	1.443 (8)	C16—H16C	0.9600
N3—C1	1.491 (7)	C17—H17A	0.9600
N4—C3	1.391 (10)	C17—H17B	0.9600
N4—C4	1.479 (10)	C17—H17C	0.9600
N5—C5	1.450 (9)	C18—H18A	0.9600
N5—C6	1.475 (9)	C18—H18B	0.9600
N6—C8	1.467 (7)	C18—H18C	0.9600
N6—C7	1.469 (6)	C19—H19A	0.9600
N7—C9	1.452 (7)	C19—H19B	0.9600
N7—C10	1.477 (8)	C19—H19C	0.9600
N8—C11	1.417 (9)	C20—H20A	0.9600
N8—C12	1.473 (8)	C20—H20B	0.9600
N9—C13	1.418 (10)	C20—H20C	0.9600
N9—C14	1.479 (10)	C21—H21A	0.9600
N10—C15	1.477 (8)	C21—H21B	0.9600
N10—C16	1.494 (9)	C21—H21C	0.9600
N11—C17	1.446 (11)	C22—H22A	0.9600
N11—C18	1.512 (12)	C22—H22B	0.9600
N12—C19	1.441 (8)	C22—H22C	0.9600
N12—C20	1.460 (8)	C23—H23A	0.9600
N13—C21	1.462 (9)	C23—H23B	0.9600
N13—C22	1.462 (8)	C23—H23C	0.9600
N14—C23	1.453 (9)	C24—H24A	0.9600
N14—C24	1.454 (10)	C24—H24B	0.9600
C1—H1A	0.9600	C24—H24C	0.9600
C1—H1B	0.9600	W1—S3	2.1936 (14)
C1—H1C	0.9600	W1—S4	2.2030 (14)
C2—H2A	0.9600	W1—S2	2.2039 (12)
C2—H2B	0.9600	W1—S1	2.2105 (14)
C2—H2C	0.9600	W1—Ag1	2.9461 (6)
C3—H3A	0.9600	W1—Ag1^i^	2.9645 (7)
C3—H3B	0.9600	Ag1—S1	2.4918 (14)
C3—H3C	0.9600	Ag1—S2	2.5698 (14)
C4—H4A	0.9600	Ag1—S4^ii^	2.6167 (16)
C4—H4B	0.9600	Ag1—S3^ii^	2.6202 (16)
C4—H4C	0.9600	Ag1—W1^ii^	2.9645 (7)
C5—H5A	0.9600	S3—Ag1^i^	2.6202 (16)
C5—H5B	0.9600	S4—Ag1^i^	2.6167 (16)
C5—H5C	0.9600		
			
O3—Sm1—O2	92.45 (12)	N5—C5—H5C	109.5
O3—Sm1—O4	88.58 (13)	H5A—C5—H5C	109.5
O2—Sm1—O4	157.31 (12)	H5B—C5—H5C	109.5
O3—Sm1—O1	158.08 (12)	N5—C6—H6A	109.5
O2—Sm1—O1	94.44 (12)	N5—C6—H6B	109.5
O4—Sm1—O1	92.95 (12)	H6A—C6—H6B	109.5
O3—Sm1—O9	128.05 (12)	N5—C6—H6C	109.5
O2—Sm1—O9	79.75 (12)	H6A—C6—H6C	109.5
O4—Sm1—O9	81.80 (13)	H6B—C6—H6C	109.5
O1—Sm1—O9	73.74 (12)	N6—C7—H7A	109.5
O3—Sm1—O8	77.56 (12)	N6—C7—H7B	109.5
O2—Sm1—O8	77.52 (12)	H7A—C7—H7B	109.5
O4—Sm1—O8	80.58 (12)	N6—C7—H7C	109.5
O1—Sm1—O8	124.27 (12)	H7A—C7—H7C	109.5
O9—Sm1—O8	50.53 (12)	H7B—C7—H7C	109.5
O3—Sm1—O6	79.60 (12)	N6—C8—H8A	109.5
O2—Sm1—O6	75.82 (12)	N6—C8—H8B	109.5
O4—Sm1—O6	126.53 (12)	H8A—C8—H8B	109.5
O1—Sm1—O6	81.97 (12)	N6—C8—H8C	109.5
O9—Sm1—O6	143.84 (12)	H8A—C8—H8C	109.5
O8—Sm1—O6	143.82 (12)	H8B—C8—H8C	109.5
O3—Sm1—O5	78.65 (12)	N7—C9—H9A	109.5
O2—Sm1—O5	126.25 (12)	N7—C9—H9B	109.5
O4—Sm1—O5	76.16 (12)	H9A—C9—H9B	109.5
O1—Sm1—O5	80.49 (12)	N7—C9—H9C	109.5
O9—Sm1—O5	144.96 (12)	H9A—C9—H9C	109.5
O8—Sm1—O5	146.92 (12)	H9B—C9—H9C	109.5
O6—Sm1—O5	50.43 (11)	N7—C10—H10A	109.5
O3—Sm1—N2	102.91 (13)	N7—C10—H10B	109.5
O2—Sm1—N2	76.03 (13)	H10A—C10—H10B	109.5
O4—Sm1—N2	81.64 (13)	N7—C10—H10C	109.5
O1—Sm1—N2	98.95 (13)	H10A—C10—H10C	109.5
O9—Sm1—N2	25.25 (12)	H10B—C10—H10C	109.5
O8—Sm1—N2	25.35 (12)	N8—C11—H11A	109.5
O6—Sm1—N2	151.82 (12)	N8—C11—H11B	109.5
O5—Sm1—N2	157.72 (12)	H11A—C11—H11B	109.5
O3—Sm1—N1	75.58 (12)	N8—C11—H11C	109.5
O2—Sm1—N1	100.93 (12)	H11A—C11—H11C	109.5
O4—Sm1—N1	101.28 (13)	H11B—C11—H11C	109.5
O1—Sm1—N1	82.68 (12)	N8—C12—H12A	109.5
O9—Sm1—N1	156.37 (12)	N8—C12—H12B	109.5
O8—Sm1—N1	153.01 (12)	H12A—C12—H12B	109.5
O6—Sm1—N1	25.26 (11)	N8—C12—H12C	109.5
O5—Sm1—N1	25.38 (12)	H12A—C12—H12C	109.5
N2—Sm1—N1	176.61 (13)	H12B—C12—H12C	109.5
O1—P1—N5	109.6 (3)	N9—C13—H13A	109.5
O1—P1—N3	108.9 (2)	N9—C13—H13B	109.5
N5—P1—N3	115.6 (3)	H13A—C13—H13B	109.5
O1—P1—N4	115.9 (3)	N9—C13—H13C	109.5
N5—P1—N4	103.3 (3)	H13A—C13—H13C	109.5
N3—P1—N4	103.6 (3)	H13B—C13—H13C	109.5
O2—P2—N8	111.1 (2)	N9—C14—H14A	109.5
O2—P2—N6	108.1 (2)	N9—C14—H14B	109.5
N8—P2—N6	109.6 (2)	H14A—C14—H14B	109.5
O2—P2—N7	111.1 (2)	N9—C14—H14C	109.5
N8—P2—N7	107.4 (3)	H14A—C14—H14C	109.5
N6—P2—N7	109.5 (2)	H14B—C14—H14C	109.5
O3—P3—N10	110.2 (3)	N10—C15—H15A	109.5
O3—P3—N11	109.9 (3)	N10—C15—H15B	109.5
N10—P3—N11	109.7 (4)	H15A—C15—H15B	109.5
O3—P3—N9	108.2 (3)	N10—C15—H15C	109.5
N10—P3—N9	109.7 (3)	H15A—C15—H15C	109.5
N11—P3—N9	109.1 (4)	H15B—C15—H15C	109.5
O4—P4—N14	119.3 (2)	N10—C16—H16A	109.5
O4—P4—N12	107.6 (2)	N10—C16—H16B	109.5
N14—P4—N12	105.3 (3)	H16A—C16—H16B	109.5
O4—P4—N13	108.0 (2)	N10—C16—H16C	109.5
N14—P4—N13	102.2 (3)	H16A—C16—H16C	109.5
N12—P4—N13	114.8 (3)	H16B—C16—H16C	109.5
P1—O1—Sm1	163.2 (2)	N11—C17—H17A	109.5
P2—O2—Sm1	167.6 (2)	N11—C17—H17B	109.5
P3—O3—Sm1	167.8 (2)	H17A—C17—H17B	109.5
P4—O4—Sm1	158.3 (2)	N11—C17—H17C	109.5
N1—O5—Sm1	96.2 (3)	H17A—C17—H17C	109.5
N1—O6—Sm1	96.4 (3)	H17B—C17—H17C	109.5
N2—O8—Sm1	96.0 (3)	N11—C18—H18A	109.5
N2—O9—Sm1	96.9 (3)	N11—C18—H18B	109.5
O7—N1—O6	121.9 (5)	H18A—C18—H18B	109.5
O7—N1—O5	122.1 (5)	N11—C18—H18C	109.5
O6—N1—O5	116.1 (4)	H18A—C18—H18C	109.5
O7—N1—Sm1	171.9 (4)	H18B—C18—H18C	109.5
O6—N1—Sm1	58.4 (2)	N12—C19—H19A	109.5
O5—N1—Sm1	58.4 (2)	N12—C19—H19B	109.5
O10—N2—O9	121.3 (5)	H19A—C19—H19B	109.5
O10—N2—O8	122.3 (5)	N12—C19—H19C	109.5
O9—N2—O8	116.3 (4)	H19A—C19—H19C	109.5
O10—N2—Sm1	174.3 (4)	H19B—C19—H19C	109.5
O9—N2—Sm1	57.9 (2)	N12—C20—H20A	109.5
O8—N2—Sm1	58.7 (2)	N12—C20—H20B	109.5
C2—N3—C1	114.1 (5)	H20A—C20—H20B	109.5
C2—N3—P1	120.9 (4)	N12—C20—H20C	109.5
C1—N3—P1	120.6 (4)	H20A—C20—H20C	109.5
C3—N4—C4	107.7 (7)	H20B—C20—H20C	109.5
C3—N4—P1	125.6 (5)	N13—C21—H21A	109.5
C4—N4—P1	121.5 (5)	N13—C21—H21B	109.5
C5—N5—C6	115.8 (6)	H21A—C21—H21B	109.5
C5—N5—P1	121.6 (5)	N13—C21—H21C	109.5
C6—N5—P1	120.3 (5)	H21A—C21—H21C	109.5
C8—N6—C7	114.5 (4)	H21B—C21—H21C	109.5
C8—N6—P2	119.8 (3)	N13—C22—H22A	109.5
C7—N6—P2	122.6 (4)	N13—C22—H22B	109.5
C9—N7—C10	114.7 (5)	H22A—C22—H22B	109.5
C9—N7—P2	125.0 (4)	N13—C22—H22C	109.5
C10—N7—P2	119.0 (4)	H22A—C22—H22C	109.5
C11—N8—C12	114.7 (6)	H22B—C22—H22C	109.5
C11—N8—P2	120.9 (5)	N14—C23—H23A	109.5
C12—N8—P2	123.8 (5)	N14—C23—H23B	109.5
C13—N9—C14	112.7 (8)	H23A—C23—H23B	109.5
C13—N9—P3	123.3 (5)	N14—C23—H23C	109.5
C14—N9—P3	123.4 (7)	H23A—C23—H23C	109.5
C15—N10—C16	115.5 (5)	H23B—C23—H23C	109.5
C15—N10—P3	119.7 (5)	N14—C24—H24A	109.5
C16—N10—P3	123.4 (5)	N14—C24—H24B	109.5
C17—N11—C18	121.9 (9)	H24A—C24—H24B	109.5
C17—N11—P3	119.2 (6)	N14—C24—H24C	109.5
C18—N11—P3	118.8 (8)	H24A—C24—H24C	109.5
C19—N12—C20	113.5 (5)	H24B—C24—H24C	109.5
C19—N12—P4	121.5 (4)	S3—W1—S4	110.05 (5)
C20—N12—P4	122.1 (4)	S3—W1—S2	108.01 (5)
C21—N13—C22	112.5 (6)	S4—W1—S2	108.55 (5)
C21—N13—P4	118.9 (4)	S3—W1—S1	108.19 (6)
C22—N13—P4	120.2 (5)	S4—W1—S1	108.69 (6)
C23—N14—C24	113.4 (7)	S2—W1—S1	113.33 (5)
C23—N14—P4	123.4 (5)	S3—W1—Ag1	125.32 (4)
C24—N14—P4	119.8 (5)	S4—W1—Ag1	124.62 (4)
N3—C1—H1A	109.5	S2—W1—Ag1	57.73 (4)
N3—C1—H1B	109.5	S1—W1—Ag1	55.61 (3)
H1A—C1—H1B	109.5	S3—W1—Ag1^i^	58.81 (4)
N3—C1—H1C	109.5	S4—W1—Ag1^i^	58.66 (4)
H1A—C1—H1C	109.5	S2—W1—Ag1^i^	148.29 (4)
H1B—C1—H1C	109.5	S1—W1—Ag1^i^	98.38 (3)
N3—C2—H2A	109.5	Ag1—W1—Ag1^i^	153.978 (9)
N3—C2—H2B	109.5	S1—Ag1—S2	93.54 (4)
H2A—C2—H2B	109.5	S1—Ag1—S4^ii^	120.91 (5)
N3—C2—H2C	109.5	S2—Ag1—S4^ii^	120.21 (5)
H2A—C2—H2C	109.5	S1—Ag1—S3^ii^	120.75 (5)
H2B—C2—H2C	109.5	S2—Ag1—S3^ii^	117.31 (5)
N4—C3—H3A	109.5	S4^ii^—Ag1—S3^ii^	86.93 (5)
N4—C3—H3B	109.5	S1—Ag1—W1	47.06 (3)
H3A—C3—H3B	109.5	S2—Ag1—W1	46.48 (3)
N4—C3—H3C	109.5	S4^ii^—Ag1—W1	137.29 (4)
H3A—C3—H3C	109.5	S3^ii^—Ag1—W1	135.70 (4)
H3B—C3—H3C	109.5	S1—Ag1—W1^ii^	151.26 (4)
N4—C4—H4A	109.5	S2—Ag1—W1^ii^	115.17 (3)
N4—C4—H4B	109.5	S4^ii^—Ag1—W1^ii^	45.97 (3)
H4A—C4—H4B	109.5	S3^ii^—Ag1—W1^ii^	45.74 (3)
N4—C4—H4C	109.5	W1—Ag1—W1^ii^	161.657 (17)
H4A—C4—H4C	109.5	W1—S1—Ag1	77.33 (4)
H4B—C4—H4C	109.5	W1—S2—Ag1	75.78 (4)
N5—C5—H5A	109.5	W1—S3—Ag1^i^	75.44 (4)
N5—C5—H5B	109.5	W1—S4—Ag1^i^	75.37 (4)
H5A—C5—H5B	109.5		

Symmetry codes: (i) *x*, −*y*+1/2, *z*−1/2; (ii) *x*, −*y*+1/2, *z*+1/2.

## References

[bb1] Cao, Y., Zhang, J.-F., Qian, J. & Zhang, C. (2007). *Acta Cryst.* E**63**, m2076–m2077.

[bb2] Niu, Y. Y., Zheng, H. G., Hou, H. W. & Xin, X. Q. (2004). *Coord. Chem. Rev.* **248**, 169–183.

[bb3] Rigaku (2007). *CrystalClear* Rigaku Corporation, Tokyo, Japan.

[bb4] Sheldrick, G. M. (2008). *Acta Cryst.* A**64**, 112–122. 10.1107/S0108767307043930 18156677

[bb5] Tang, G., Zhang, J. & Zhang, C. (2008). *Acta Cryst.* E**64**, m478. 10.1107/S1600536808001256 PMC2960880 21201866

[bb6] Tang, G., Zhang, J., Zhang, C. & Lu, L. (2008). *Acta Cryst.* E**64**, m399–m400. 10.1107/S1600536807066597 PMC2960413 21201349

[bb7] Zhang, J. (2010). *Acta Cryst.* E**66**, m1479. 10.1107/S1600536810042728 PMC3009021 21588894

[bb8] Zhang, J. (2011). *Acta Cryst.* E**67**, m1206–m1207. 10.1107/S1600536811030996 PMC3200717 22065643

[bb9] Zhang, J.-F., Cao, Y., Qian, J. & Zhang, C. (2007). *Acta Cryst.* E**63**, m2248–m2249.

[bb10] Zhang, J., Qian, J., Cao, Y. & Zhang, C. (2007). *Acta Cryst.* E**63**, m2386–m2387.

[bb11] Zhang, C., Song, Y. L. & Wang, X. (2007). *Coord. Chem. Rev.* **251**, 111–141.

